# Representing Route Familiarity Using the Abstraction Hierarchy Framework

**DOI:** 10.3390/geriatrics6030081

**Published:** 2021-08-19

**Authors:** Rashmi P. Payyanadan, John D. Lee

**Affiliations:** 1 Touchstone Evaluations, Detroit, MI 48202, USA; 2 Department of Industrial and Systems Engineering, University of Wisconsin-Madison, Madison, WI 53706, USA; john.d.lee@wisc.edu

**Keywords:** route familiarity, older drivers, abstraction hierarchy, route choice

## Abstract

Familiarity with a route is influenced by levels of dynamic and static knowledge about the route and the route network such as type of roads, infrastructure, traffic conditions, purpose of travel, weather, departure time, etc. To better understand and develop route choice models that can incorporate more meaningful representations of route familiarity, OBDII devices were installed in the vehicles of 32 drivers, 65 years and older, for a period of three months. Personalized web-based trip diaries were used to provide older drivers with post-trip feedback reports about their risky driving behaviors, and collect feedback about their route familiarity, preferences, and reasons for choosing the route driven vs. an alternate low-risk route. Feedback responses were analyzed and mapped onto an abstraction hierarchy framework, which showed that among older drivers, route familiarity depends not only on higher abstraction levels such as trip goals, purpose, and driving strategies, but also on the lower levels of demand on driving skills, and characteristics of road type. Additionally, gender differences were identified at the lower levels of the familiarity abstraction model, especially for driving challenges and the driving environment. Results from the analyses helped highlight the multi-faceted nature of route familiarity, which can be used to build the necessary levels of granularity for modelling and interpretation of spatial and contextual route choice recommendation systems for specific population groups such as older drivers.

## 1. Introduction

A number of studies have established the role of familiarity as an important factor for driving. Familiarity is known to govern route choice [1] and there is a growing need for familiarity to be included in choice models. In the driving domain, familiarity is commonly assessed by either the expectancy of the driver (travel time, travel distance, traffic conditions, weather), prior driving experience with the route (knowledge of the route attributes, road characteristics, driving environment), personal characteristics of the driver (socioeconomic, choice preference), trip characteristics (purpose, time of day), or the referent frequencies of the route learnt by driving the same routes. A review of the common approaches used to assess route familiarity is shown in Table 1.

Until recently, most studies relied on simulator environments due to the technology limitations and costs associated with capturing and assessing large naturalistic driving data. Hence, Table 1 includes both simulator and on-road studies used to assess route familiarity.

Table 1 shows that although these studies have provided important contributions in understanding route familiarity, a major drawback is that the construct of familiarity is broadly defined by ‘the frequency of exposure to an item’, or by ‘the opportunity to learn about the item’; determined through characteristics of the item and frequency of exposure to the item [17]. Hence different studies capture different aspects of route familiarity. Additionally, many of the studies assess route familiarity using simulated environments. Although simulator studies use measurement validity—defined by indicators that measure the concept of interest to determine whether inferences about real driving behaviors can be made from driving simulator data [18]; simulator studies may not necessarily capture the construct of familiarity. This is because approaches implemented to establish route familiarity in simulator settings involve training drivers on preset routes, and using questionnaires and retrieval cues. However, extensive work by Yonelinas [19,20] has provided empirical evidence to suggest that learning novel associations such as a new route is associated with the construct of recollection; whereas the construct of familiarity only supports novel learning under very limited conditions. Thus, studies that train drivers over a short period of time on preset routes, often capture responses that mainly reflect recollection—conscious experience of remembering associated with when or where an item was studied; versus familiarity—conscious experience of knowing, which cannot necessarily discriminate when or where an item was studied [19,20,21].

For older drivers in particular, route familiarity plays an important role in prolonging their mobility and driving safety. Older adults are generally safe drivers. However, driving maneuvers such as left turns and negotiating intersections can become more challenging with age [22,23]. When driving conditions become challenging—especially due to declining health, it can affect driving ability. Under such conditions, older drivers are known to self-regulate by avoiding rush hours, intersections, night-time driving, bad weather, and driving on unfamiliar roads [24].

Driving on familiar roads as a self-regulation strategy has been shown to improve the driving safety outcomes of older drivers. A study on intersection negotiation problems found that drivers 60 years and older conducted fewer unsafe lane change maneuvers, left-turn errors, and hard braking events before lane change on familiar versus unfamiliar routes [25]. Older drivers with Parkinson’s disease have been shown to have fewer safety errors for turn taking and speed control on familiar routes [26], and less likely to make navigational errors and get lost during wayfinding on familiar versus unfamiliar routes [27].

However, only limited research has been conducted on understanding what factors contribute to route familiarity among older drivers. Work by Zhang and Levinson [28] showed that compared to younger drivers, older drivers tend to prefer routes with similar trip attributes such as travel time, distance, traffic delays, and speed limits for commuting to events and visiting friends and family, but not for shopping and recreational trips. Furthermore, compared to younger drivers, older drivers prefer to drive routes that they are familiar with because familiarity with the route increased recall of the environment and its objects [29], and reduced the likelihood of getting lost [30].

Although older drivers prefer familiar routes, few navigation systems consider familiarity as part of the route planning feature. Current navigation systems implement route choice algorithms mainly for obtaining shortest route [31], routes with less navigational complexity at intersections [32], simplest route [33], scenic routes [34], regionalized path planning [35], landmarks [36], and traffic congestion [37]. Some navigational systems have implemented multi-criteria route selection based on driver preferences [38] but fall short as they are primarily focused on minimizing costs such as time and distance, rather than balancing factors such as familiarity, fewer turns, and stop lights [39]. Thus, considering familiarity as part of a multi-criteria route selection algorithm could benefit the wayfinding and route planning needs of older drivers in particular, and prolong their mobility and driving safety.

However, modelling route choice as a function of familiarity is complex as it involves a number of factors based on the driver’s preference, their cognitive map, and characteristics of the driving environment [40]. Given the limited understanding on the influence of familiarity on route choice especially among older adults, the goal of this study is to determine the different factors that contribute to route familiarity using naturalistic driving data and familiarity feedback responses collected from 29 drivers, 65 years and older, for a period of three months. With the continued advances in driver support, navigation, driver monitoring, and safety management systems; a better understanding of the factors that contribute to route familiarity can provide opportunities to customize in-vehicle systems that can support the delivery of relevant routing options, driving safety, and behavioral feedback to meet the specific driving challenges, safety needs, and driving preferences of drivers, especially older drivers.

## 2. Using Abstraction Hierarchy to Represent Route Familiarity

Abstraction hierarchy models have long been used to represent categories of mental representations of object or events abstracted from observations of the environment [41]. However, not all categories observed in the environment can be structured into hierarchies. For example, Rosch et al. [42] showed that for some concepts such as ‘concreteness’ or ‘imageability’, there does not exist a preference for basic, subordinate, and superordinate levels of categorization. Whereas other studies have shown that only the basic levels are representational of concepts [43]. For understanding familiarity, early work using abstraction hierarchy models have shown that familiarity is strongly associated with categorization of features to describe an item or event as familiar [44]. Thus, abstraction hierarchies serve as a suitable framework for describing the features of familiarity that influence decision making like route choice.

### 2.1. Rasmussen’s Abstraction Hierarchy Framework

In cognitive engineering, Rasmussen’s abstraction hierarchy (AH) framework has been extensively used to represent systems in a way that reflects human memory and problem-solving characteristics, but is also event and actor independent, allowing for a wide range of situations and unforeseen events [45]. Such a framework offers an opportunity to model the functional structure of the physical, social, and cultural environments of actors in the system. This enables (a) identifying constraints on actors, (b) revealing possibilities of actions available, (c) determining rationale for actor’s behavior, (d) application across a range of situations, and (e) design outcomes that can support the actor in dealing with a variety of events [46,47]. Thus, Rasmussen’s AH model serves as a useful theoretical framework for representing route familiarity because it provides a systematic description of the system in engineering terms that is compatible with the psychological representation people use to deal with complex systems [45].

Rasmussen’s AH framework is commonly used for modelling the functional structure of the environment—also referred to as the abstraction–decomposition space and is represented as a matrix (Figure 1). In the abstraction–decomposition space (Figure 1), the vertical axis comprises of the means-end relations of the abstraction dimension, and the horizontal axis comprises of the part–whole relations of the decomposition dimension [46]. The cells of the matrix are populated with representations of the functional structure of the environment and is specific to the particular level of abstraction and decomposition. While the abstraction dimension is typically described by five levels; there are no set levels for the decomposition dimension.

The abstraction dimension describes the properties of the environment used for achieving an end and is comprised of five levels reflecting distinct concepts: functional purpose, abstract functions, generalized functions, physical functions, and physical form. Functional purpose represents the primary purpose of the system; abstract function represents the intentions or the intended operational state of the system; and generalized functions represent the functional relationships in the system independent of physical manifestation. Lastly, the physical function level represents the mechanical, electrical, or chemical processes of the system or its parts; and the physical form represents the appearance or configuration of the systems and its parts. Moving up or down the levels of abstraction represent the means-end relations. The abstraction dimension allows both a bottoms-up approach for describing the use of the system components and functions for serving the purpose or goal; and a top-down approach of how the purpose or goal can be implemented by the functions and components of the system. Although each cell can be populated with specific constraints or means, it is not considered efficient [48]. Instead, it is recommended that only the constraints or means that provide meaningful or useful information about the environment be incorporated into the abstraction-decomposition matrix–represented along the diagonal of the matrix [46].

Each level of the abstraction dimension describes a different set of constraints or means associated with the activities in a system. For example, to drive to the hospital for a doctor’s appointment, a driver may choose a route that has less traffic, fewer stop lights, and park in a spot that is easy to access [49]. Here the route chosen, and parking spot are the means for getting to the doctor’s appointment on time, and the traffic and stop lights are the constraints of the driving environment on the driver. These means-end relations are determined as a how–what–why triad, representing the demands on the actor and the context of the situation for the actor [46,47,48,49,50].

The decomposition dimension consists of few or many levels representing details of the functional environment as its parts and wholes. Thus, the levels of decomposition are connected by part-whole relations, where the lower levels are the functional parts of the higher levels, and the higher levels are the wholes of the lower levels—representing different levels of the same system.

### 2.2. Levels within Rasmussen’s Abstraction Hierarchy Framework

The abstraction dimension (Figure 1) is made up of five qualitatively distinct concepts used for modeling the structural properties of the environment and is characterized by the means-ends relations. The labels ascribed to each level and the constraints or means represented within each level by Rasmussen [50] are defined below:

Functional purpose level—The function purpose level represents the overall goals and purpose of the system, objectives, and the external limits on the system due to the environment. The system’s purpose remains relatively constant, while the objectives and external limits of a system are dynamic, changing with respect to the situation [51]. The system can have multiple objectives. The external limits refer to the properties of the environment that impose on the system’s purpose [46]. At the functional purpose level, purpose, objectives, and external limits govern the interaction between the system and the environment.

Abstract function level—The abstract function level represents the values and priority measures needed to fulfill the purpose of the system [46]. Criteria such as selecting the shortest route, selecting the fastest route, etc., can allow the driver to compare, prioritize, and allocate resources to achieve the trip purpose. Assessing these criteria can help evaluate whether the purpose is fulfilled.

Generalized function level—The generalized function level represents the functions that must be supported to fulfill the system’s purpose, independent of the underlying physical objects or object-related processes needed to implement them [46]. This level includes challenges such as maintaining a certain speed and acceleration with other drivers on the road, overtake vehicles if they are driving to slow, etc. [52]. While there are no reported variations on how factors at this level are characterized, these functions are represented in general terms using terminology common to the field, such that the functions indicate the type of system but not the specific system [46,47,48,49,50,51,52,53].

Physical function level—The physical function level represents the object-related processes or parts of the system that are used to characterize the functional states [50]. The object-related processes or parts are tightly related to the physical objects, and represented by their reason for use, or by their limiting properties. The resolution of the details represented in this level depends on the specific task or interaction with the system. For a trip, the number of stop signs, street parking, etc. (physical functions level), influences the purpose-related functions such as speed maintenance, start–stop events, etc. (generalized functions level), affecting the evaluation criteria such as duration of travel (abstract function level), and the goals and objectives of the trip such as reaching on time at the destination (functional purpose level). The physical representation is tightly coupled with the functional states, where changes at the physical functional level propagate up the hierarchy and influence the higher levels [50].

Physical form level—The bottommost level represents the physical appearance and configuration of the system and its parts [50]. Representation of the system and its parts at this level reflects what parts are vital for interaction with, and manipulation of the system to achieve the purpose-related functions of the system. This level is represented by names or attributes that can help identify and distinguish objects and their properties for navigating the system [50].

## 3. Method

To determine the different factors within the abstraction hierarchy levels that contribute to route familiarity among older drivers, 32 drivers, 65 years and older, were recruited for the study. The participant vehicles were instrumented with OBDII devices, and data from the devices were fed into a customized web-based trip diary to provide drivers with feedback about their trips driven, risky driving behaviors such as speeding and hard braking events, and alternate low-risk routes. For each trip driven, participants were asked to provide feedback about their route familiarity and reasons for preferring the driven vs. the alternate low-risk route suggested.

### 3.1. Participants

A total of 32 drivers, 65 years and older, were recruited from a larger study focused on understanding the needs of older adults at risk of entering nursing care [54]. Participant’s age ranged from 65 to 82 years. Study inclusion criteria required that participants were 65 years or older, and in the last 12 months had experienced either a fall, felt sad or depressed, received home-health services, stayed in a skilled nursing facility, visited the emergency room, or was admitted to the hospital. Additionally, study eligibility also required that participants hold a valid driver’s license, own their own vehicle, and drive at least twice a week. Participants were excluded if there were living in a hospice center, assisted living facility without access to a stove, in a nursing home, or needed help getting in and out of a bed or chair.

Although naturalistic driving data was collected from the 32 participants, only 29 participants provided feedback responses about their route familiarity. Thus, the analysis was conducted using only data from the 29 participants. The demographic data along with the trip details of the 29 participants in the study are shown in Table 2.

### 3.2. OBDII Devices

Geotab GO6 OBDII devices were installed in the vehicles of 32 older drivers for three months to collect trip information using GPS data. The Geotab GO6 OBDII devices recorded risky driving behaviors such as speeding, hard braking, accelerating, and cornering events. Due to the limitations of the OBDII device recording capabilities, the risky driving behaviors were recorded as events, where event-based measures reflect only the frequency of the behavior. Participants were given access to their data using web-based trip diaries to receive feedback about their trips, driving behaviors, and collect feedback responses about their trip familiarity.

### 3.3. Trip Diaries

Web-based trip diaries were used to provide information about the participant’s trips driven and risky driving behavior events, along with alternate low-risk route options. The suggested low-risk route option is a route with the least number of left turns, U-turns, traffic incidents, and lane closures among a subset of routes for any given origin and destination, with minimal cost to trip distance and travel time [49]. Participants were requested to access their trip diary page two to three times a week, review each of their trips driven, risky driving behavior events, alternate low-risk route suggestions, and provide feedback responses to the three familiarity questions for each trip driven.

The three familiarity questions were: (a) This route is familiar to me (referring to the driven route), (b) the alternate suggested route is familiar to me (referring to the low-risk alternate route), and (c) Why did you choose the driven route? The first two familiarity questions: familiarity with the route driven and familiarity with the alternate low-risk suggested route, were yes/no questions. The third question on reasons for choosing a route was an open-ended question where participants could state the decisions and challenges that influenced their route choice.

### 3.4. Developing a Familiarity Abstraction Hierarchy Framework

A total of 612 familiarity feedback responses from the trip diary were recorded from older drivers over the three-month period. Concepts related to route familiarity were extracted from the open-ended familiarity responses using prompts and keywords developed by Naikar [46]. These concepts were to populate the different levels of the abstraction hierarchy framework representing route familiarity (shown in Table 3).

Table 3 provides a guide to the kinds of properties that should be searched for to develop an abstraction dimension. For example, the ‘prompts’ in Table 3 for the functional purpose level of abstraction indicate that the information should reflect aspects such as the high-level objectives or ultimate purpose of the system, needs of the environment, and what the system has been designed to achieve. Whereas the ‘keywords’ at this level of abstraction indicate the different ways in which the purposes and external constraints may be revealed in a system. For example, motives, view, and rationale (high level purposes), or laws, regulations, and limits (external constraints).

The abstraction hierarchy representing route familiarity was then reviewed and refined through an iterative process by four subject matter experts that have extensive experience in road safety research as well as applied systems thinking research across a range of domains. Each level of the abstraction hierarchy was systematically reviewed and verified; and factors representing route familiarity were either agreed upon, modified, or removed if all the experts did not agree. The iterative process involved 12 iterations upon which there was agreement regarding the abstraction hierarchy content.

## 4. Results

The results are organized into two sections. The first provides a summary of the trip details and routes driven by older drivers in the study for a period of three months. The second section shows the different factors that contributed to choosing a familiar route among older drivers.

### 4.1. Trips Driven by Older Drivers

A total of 5365 trips were driven by older drivers during the three-month period. Older drivers’ trips were on average 7.1 miles long, had an average trip time of 13.2 min, and mean speed of 23.4 miles/h. A summary of the risky driving behavior events recorded from the OBDII device across the trips driven during the three-month period is shown in Table 4.

Familiarity responses were received for 5.3% of the trips driven. Based on the trips with familiarity responses, 78% of the driven routes were familiar (CI [74.9, 82.1]), 14% unfamiliar [10.3, 17.5]), and the remaining 8% had no responses (CI [4.3, 11.6]). A paired sample *t*-test was conducted on the risky driving behavior events per mile driven by older drivers on the familiar and unfamiliar routes driven (Table 5).

Results in Table 5 showed that while there were more hard acceleration events and that the overall risky events were higher on unfamiliar routes; they were not significant. The lack of significant differences in the number of risky driving behaviors events that occurred per mile driven between routes that were familiar and unfamiliar provides evidence to suggests that there was no difference in how older adults drove on routes that were familiar and unfamiliar. This indicates that there are other factors that govern why older drivers prefer familiar routes.

### 4.2. Factors That Characterize Route Familiarity among Older Drivers

**Figure 2:**
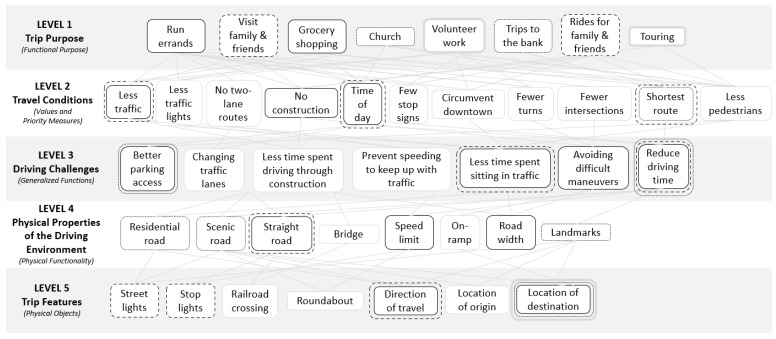
Summary of the route familiarity abstraction hierarchy framework.

A total of 612 familiarity feedback responses were recorded from older drivers during the three-month period. Familiarity feedback responses showed that seventy-six percent of the older drivers preferred the familiar route versus an alternate low-risk route, irrespective of fewer number of left turns, U-turns, travel time, and distance. Furthermore, driving familiar routes versus alternate unfamiliar low-risk routes was preferred when running errands, grocery shopping, giving rides and visiting family and friends. Naikar’s prompts and keywords (Table 3) were used to extract relevant factors that characterized route familiarity among older drivers for each level of the abstraction hierarchy. The complete abstraction hierarchy representing route familiarity is shown in Figure 2.

Each of the abstraction levels shown in Figure 2 represent five distinct levels of route familiarity: *trip purpose level* (functional purposes), *travel conditions level* (values and priority measures), *driving challenges level* (generalized functions), *physical properties of the driving environment level* (physical functionality), and *trip features level* (physical objects). Within the abstraction hierarchy, the lines connecting nodes between each abstraction level are the ‘means-end links’ that represent the why–what–how relationship. Thus, each node is the ‘what’ node that provides an understanding of the factors linked above in the hierarchy as to ‘why’ they are necessary, and the factors linked below in the hierarchy represent ‘how’ the node is achieved [47].

Familiarity feedback responses revealed eight distinct *trip purposes* (Level 1) when older drivers preferred driving familiar routes vs. alternate low risk routes. These eight distinct trip purposes were running errands with multiple stops, visiting family and friends nearby, grocery shopping, going to church, conducting volunteer work for the community, offering rides, and touring. Among these trip purposes, running errands (single trip with multiple stops to the post office, store, etc.) and grocery shopping—represented by boxes with solid black outline in Figure 2—had the most similar means-end links. This is unsurprising since running multiple errands and grocery shopping are routine activities to known destinations, and thus the *travel conditions* (Level 2) that encompass the criteria and measures to achieve the trip purpose were found to be the same, such as driving routes that had less traffic and construction, driving the shortest route, and driving during a specific time of day. To achieve the trip purpose, four *driving challenges* (Level 3) that were often overcome and stated by older drivers as an important factor for preferring a familiar route were: better parking access at the destination (easier to park and choose a close by parking spot based on direction of entry into the parking lot that the route enables), less time spent in traffic, reduced exposure to difficult driving maneuvers (often due to heavy traffic), and reduced overall time spent driving. Lastly, five *physical properties of the driving environment* (Level 4) and *trip features* (Level 5) identified for the familiar routes driven to achieve the trip purposes were that the roads were often straight and wide, had posted speed limits, and was optimal for the direction of travel and location of the destination.

Two other sets of *trip purposes* (Level 1) also had similar means-end links: visiting and giving rides to family and friends (boxes with dashed black outline in Figure 2) and driving to church and the bank (boxes with dotted black outline in Figure 2). Although volunteer work and touring (going on drives to less known areas in their county, region, or state) were also unique *trip purposes* that were identified; the factors that characterized the type of familiar route chosen at the lower abstraction levels were less obvious. This could be attributed to the fact that the destination location for both these types of *trip purposes* were not always known to the older driver, and hence the type of familiar route driven for these trip purposes is more ambiguous.

The theoretical roots for developing an abstraction hierarchy are event- and actor-independent [46]. Thus, the development of the abstraction hierarchy is focused on identifying the constraints of the system, the impact different constraints have on behavior, and how different constraints can be modified to enable safe and efficient performance or outcomes without specifying the actors who operate within the system. However, it is useful to highlight that although the eight unique *trip purposes* (Level 1) were conducted by both male and female older drivers; older male drivers were more likely to engage in volunteer work and touring trips compared to older female drivers. Additionally, at the lower levels of the abstraction hierarchy, older female drivers often stated shortest route and time of day (Level 2); avoiding complex driving maneuvers and adjusting their speed (Level 3); and taking the scenic route (Level 4) when describing the reasons they prefer a familiar route. Whereas older male drivers often drove their familiar routes because it had less traffic, less construction, and fewer pedestrians (Level 2); considered parking availability, avoiding construction, and reducing drive time (Level 3); and posted speed limits, on-ramp, and bridge use when describing the reasons they prefer their familiar route. Table 6 shows the final route familiarity abstraction hierarchy framework with the decomposition space, where the part-whole abstraction aggregates each level of the means-end abstraction by representing a familiar trip as a sequence of segments, series of maneuvers, micro adjustments of lateral and longitudinal control, and cardinality.

## 5. Discussion

Assessing route familiarity is challenging due to the number of ways familiarity manifests itself based on individual experiences accumulated over time. Older drivers in particular are a unique population group that have been driving for a better part of their life and are known to have a complex set of specific route preferences such as driving on freeways [55], driving during specific times of the day [56], and driving routes with less traffic and intersections. Yet little is known about the set of factors that characterizes a familiar route among older drivers. Understanding the different components of route familiarity and how they influence each other in a driver’s decision to choose a route could be leveraged to develop road safety interventions that are specific to the needs of older drivers. Thus, in this study, an abstraction hierarchy model was developed to identify the different factors that contribute to route familiarity, based on the post-trip feedback responses collected from older drivers about their route choice and preferences.

Feedback responses from older drivers on factors that characterized the familiar routes driven showed that driving familiar routes not only depended on higher levels of trip goals and purposes, but also on lower levels of demand on driving skills, and characteristics of road type. Additionally, as emerged in this study, there were no predetermined set of indicators for route familiarity; but there were common factors that characterized route familiarity when trip purposes were similar. Thus, the familiarity abstraction hierarchy reflected a range of contributory factors across different trip types that potentially play a role in route choice among older drivers.

As the abstraction hierarchy model is actor- and event-independent, few of the factors identified relate to individual road users, although gender differences were identified at the lower abstraction levels especially for driving challenges (Level 3) and driving environment (Level 4). These results support previous work on self-regulation strategies and preferences of older drivers [49,57,58,59], suggesting that there are a range of situations older drivers avoid, which weigh greater than those that are strictly assessed through crash risk outcomes and risky driving behaviors, and questionnaires such as the Driving Behavior Questionnaire, Driving Habits Questionnaire, and Driving Mobility Questionnaire.

The work presented in this study has attempted to establish the multi-criteria nature of route familiarity especially among older drivers. The usefulness of developing such an abstraction hierarchy framework for representing route familiarity is that it can also be operationalized to help better predict route choice for route planning and navigation systems that can better match the needs and preferences of select cohort of drivers. The following section attempts to propose an approach to operationalize the levels of route familiarity as identified by the familiarity abstraction hierarchy framework.

### 5.1. Moving from a Conceptual Framework to an Operational Measure of Route Familiarity

Most route choice models use some measure of recency, frequency, or both to understand and predict route choice behavior. In the literature, the common independent dimensions used to measure familiarity are similarity, recency, and frequency [49,50,51]—where similarity is determined by the relatedness between the characterizing attributes of two or more events [52]; recency is determined by the distribution of occurrences across time of the event; and frequency is determined by the rate of occurrences of the event [53]. In the driving domain, similar parallels to understanding wayfinding on familiar and unfamiliar routes have been implemented; where similarity measures are used to assess differences in the routes driven [54], and recency and frequency measures to assess driving tasks and activities [60].

In the familiarity abstraction hierarchy model, where the lower levels represent the physical features, processes, and attributes of a familiar route; and the higher levels represent the trip purpose and driving functions for a familiar route–similarity, recency, and frequency dimensions across the abstraction levels can be used to develop a more robust measure of route familiarity (approach proposed in Table 7).

Then for the familiarity abstraction hierarchy framework, route familiarity can be operationalized as the degree of similarity between routes, and the recency and frequency with which the routes are driven; where similarity is characterized by the degree of relatedness between shared features of two or more routes, and relatedness is the measure of overlap or distance between the two features. Further, frequency and recency are represented in the structure of the five levels of the similarity measure and the reference set of routes upon which they are defined. Thus, a measure of route familiarity that captures the five levels of familiarity can be represented as a function of the similarity between trip purposes, travel conditions, driving challenges, properties of the driving environment, and trip features.

### 5.2. Utility and Challenges of the Familiarity Abstraction Hierarchy Framework

In the familiarity abstraction hierarchy framework, assumptions are made about stated familiarity, and the observed (route driven, direction of travel, sequence of events) and unobserved (purpose, constraints) activities of the driver—represented as features within levels of the framework. An important challenge for such a framework is that the probability of a feature being a member of a particular abstraction level to describe familiarity is mainly judged on the basis of how representative the feature is of the level [55]. For example, features such as fastest and safest route, although not directly associated with route familiarity, represent criteria chosen by older adults as reasons for preferring a familiar route. Thus, the framework does not require that every input into each of the abstraction levels be a direct feature of route familiarity. Rather it has a less restrictive requirement in that the inputs at each level simply be associated with familiarity. Additionally, the goal of the framework is to estimate route familiarity using naturalistic driving data. This can place a high demand on data validation and may bias the base rate probabilities of the representations within a familiarity level [56]. Lastly, the abstraction hierarchy levels are fixed, creating a stable environment for parameter estimation and implementing value learning algorithms; but suffers from increased computational complexity. Whereas adaptive abstractions can enable algorithms to find abstraction levels that can adapt to the current state, or by aggregating parts of the model by grouping levels–allowing model minimization [57].

There are a number of limitations that arise from the availability or lack of information. Information needed for developing such multi-level models can be greatly limited by the sensor technology suite, affecting the reliability, usability, and integrity of the information needed for capturing the different feature representations within the abstraction levels [58]. However, there are also concerns that arise as more information is added to the representations, and as they become more specific within each of the abstraction levels. Firstly, it can produce high specificity among the features that might be unrelated to the baseline preference of the user, decrease the meaningfulness of the feature, and result in choices that are inconsistent with user preferences [59]. Secondly, increased representations and feature complexity can bias analyses measuring the strength of similarity on the familiarity levels. Thirdly, greater representations can lead to shared attributes resulting in cross-correlation. This in turn can lead to erratic coefficient estimation and dilute the robustness of estimating the effect of individual features of the model on familiarity. Additionally, recent work has highlighted the limited understanding of whether the source of the variances from individual-specific constructs such as familiarity and route choice are from preference heterogeneity or process heterogeneity. Where preference heterogeneity refers to the different preferences based on the context, situation, or experience; and process heterogeneity refers to the different decision rules used to implement the preference attributes [61]. Future work could shed light on how often these challenges may occur in practice.

Lastly, a number of features are incorporated within the levels of the familiarity abstraction hierarchy framework such as directionality of the trip, purpose, etc. However, there is no clear understanding of the order or ranking of these criteria within each of the abstraction levels, and whether order is important. While the proposed operationalization of the familiarity abstraction hierarchy framework provides structure for incorporating ranking of criteria and estimating its importance; determining ranking or ordering is needed, and future work could address the importance of ranking, and under what driving context and conditions. Studies conducted on ranking features or categories within abstraction levels has suggested increased dependency of the representations on the geographic context, thereby reducing the significance of the user’s overall ranking and context [62]. Additionally, the representations captured from the feedback and preferences of older drivers are of varying generality (such as ‘safe’, ‘interesting’, etc.), which could be split into several lower-level attributes as well. Future work can explore better ways to characterize user feedback and description of preferences.

## 6. Conclusions

The familiarity abstraction hierarchy framework provides a useful first step towards a more meaningful measure of route familiarity and capturing the challenges faced by different types of drivers. Such a framework of route familiarity could help guide the development of better route choice models that can be customized to meet the specific route choice and safety needs of drivers. Further, the implementation of such route choice models in driver support systems can have multiple benefits, such as the ability to include familiarity in the cost function for selecting routes so that the resulting routes are more likely to be accepted; used to update membership functions and ‘if-then’ rules in different scenarios based on the driver’s route familiarity; and to assist in both pre-trip planning and dynamic route choice feedback.

## Figures and Tables

**Figure 1:**
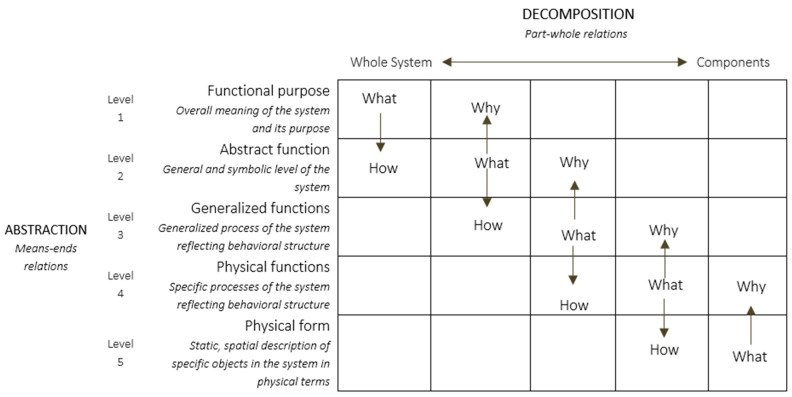
The abstraction–decomposition space.

**Table 1:** Commonly used approaches for capturing route familiarity in simulator and on-road studies.

Author	Study Goal	Study Setting	Route Familiarity Determined by
Mourant and Rockwell [2]	Effect of route familiarity on driver search and scan behavior	Simulator	Training drivers on a preset route
Allen et al. [3]	Effect of navigation system characteristics on driver route diversion behavior	Simulator	Recruiting drivers with knowledge of the road network
Kantowitz, Hanowski, and Kantowitz [4]	Effect of familiarity on the use and acceptance of ATIS	Simulator	Recruiting drivers with knowledge of the road network
Lotan [5]	Effects of network familiarity on route choice behavior	Simulator	Using travel behavior surveys and interviewing drivers with knowledge of the road network
Beijer, Smiley, and Eizenman [6]	Effect of route familiarity on glance behavior	On-road (Preset route on expressway)	Frequency of travel along a 6 km stretch of an expressway
Srinivas and Hirtle [7]	Preferences of schematized direction for familiar and unfamiliar routes	On-road (Preset route)	Training on set routes
Martens and Fox [8]	Effect of route familiarity on eye fixation changes	Simulator	Repeated exposure to the simulated driving scenarios
Uc et al. [9]	Effect of route familiarity on navigation and safety errors	On-road (Preset route)	Asking drivers of their knowledge of the road network
Mader et al. [10]	Effect of route familiarity on attention and perception processes	Simulator	Repeated exposure to the simulated driving scenarios
Yanko and Spalek [11]	Effect of route familiarity on hazard avoidance	Simulator	Training drivers on a preset route
Li, Miwa, and Morikawa [12]	Effect of familiarity to origin and destination (O-D) pairs	On-road (route known to participants)	Frequency of trips for the same O-D pair
Ramachandran, Karpov, Gupta, and Raux [13]	Modelling familiarity for navigation	On-road (preset route)	Categorizing a route as familiar if the driver can complete a route from A to B with minimal map/device assistance
Marquez et al. [14]	Effect of route familiarity on wayfinding	On-road (preset route)	Older drivers with knowledge of the road network
Payyanadan and Lee [15]	Effect of route familiarity on driving behavior and risk	On-road (daily routes driven by participants)	Administering post-trip surveys about the drivers route familiarity
Intini [16]	Effect of route familiarity on driving behavior	On-road	Recruiting drivers with knowledge of the set route

**Table 2:** Mean and standard deviation (SD) of demographic data (*n* = 29) broken down by gender.

Gender	Total	Average (SD) Age	Average (SD) Trip Distance (miles)	Average (SD) Trip Time (min)
Females	15	70 (4.6)	7.0 (2.8)	13.2 (6.7)
Males	14	73 (4.7)	7.9 (3.1)	13.6 (6.9)

**Table 3:** Example showing how the prompts and keywords developed by Naikar was used to build an abstraction hierarchy for route familiarity from the familiarity feedback responses provided by older drivers.

Prompts (*Naikar, 2013*)	Keywords (*Naikar, 2013*)	Examples of Feedback Responses for Choosing a Familiar Route	AH Levels (*Rasmussen, 1986*)
Purposes: - Why does the system exist? - Why is the system necessary? - What objectives is the system designed to achieve? External Constraints: - What type of constraints does the environment impose on the system? - What values does the environment impose on the system? - What laws and regulations do the environment impose on the system?	Purposes: Reasons, goals, objectives, aims, intentions, mission, plans, services. External constraints: laws, regulations, guidance, standards.	“Had to visit the bank, then drop off some mail, get my groceries”	LEVEL 1 Functional purpose
- What criteria can be used to determine whether the system is achieving its purposes? - What criteria can be used to judge whether the system is satisfying its external constraints? - What criteria can be used to compare the results or effects of the purpose-related functions on the functional purposes?	Criteria, measures, judgements, schedules, outcomes, limits. Measures of: effectiveness, efficiency, reliability, risk, resources, time, quality, frequency, success. Values: laws, regulations, standards, principles.	“There was construction there…had detours, needed a lot lane changes, and merging with traffic”	LEVEL 2 Values and priority measures
- What functions are required to achieve the purposes of the system? - What functions are required to satisfy the external constraints on the system? - What functions are performed in the system?	Functions, roles, responsibilities, purposes, tasks, duties, occupations.	“Higher speed limit on this route”	LEVEL 3 Purpose- related functions
- What can the physical objects in the system do or afford? - What are the functional capabilities and limitations of physical objects in the system? - What functionality is required in the system to enable the purpose-related functions?	Applications, functionality, characteristics, capabilities, limitations, capacity.	“Prefer to avoid that route because of the roundabout”	LEVEL 4 Object-related processes
- What are the physical objects in the system? - What physical objects are necessary to enable the processes and functions of the system? - What is the topography of physical objects in the system?	Objects: buildings, facilities, premises, infrastructure, fixtures, people, terrain, land, meteorological features. Material characteristics: appearance, shape, dimensions, attributes, configuration. Topography: location, layout, spacing, positions, orientations.	“Needed to leave from my friend’s place at 6PM and take the highway”	LEVEL 5 Physical objects

**Table 4:** Summary of the risky driving behavior events recorded from the OBDII device across all trips taken by older drivers during the three-month period.

Percent of Trips Driven by Older Drivers With							
**Total Drivers**	Total Trips	Average Speed (mph)	Speed Violation Events	Hard Braking Events	Hard Cornering Events	Hard Acceleration Events	Seatbelt Violation Events
29	5365	23.34	44.00	0.49	5.00	91.00	0.40

**Table 5:** Paired sample *t*-test was conducted on the risky driving behavior events per mile driven by older driver on familiar and unfamiliar routes.

Risky Driving Behavior Events/mile	For Familiar Routes (Mean)	For Unfamiliar Routes (Mean)	Estimated Difference of Familiar and Unfamiliar Routes	*p*-Value	95% CI
Harsh cornering	0.005	0.001	0.004	0.41	[−0.005, 0.013]
Hard braking	0.00	0.00	0.00	0.33	[0.000, 0.001]
Hard acceleration	2.10	3.91	−1.90	0.12	[−3.94, 0.14]
Speeding	0.87	0.78	0.09	0.74	[−0.37, 0.54]
Overall events	2.89	4.69	−1.81	0.13	[−3.76, 0.15]
Harsh cornering	0.005	0.001	0.004	0.41	[−0.005, 0.013]

**Table 6:** Levels of the abstraction hierarchy for capturing the multi-faceted factors of route familiarity.

Abstraction Dimension	Decomposition Dimension				
	Type of Trip	Sequence of Segments of a Trip	Series of Maneuvers along a Trip	Micro Adjustments of Lateral and Longitudinal Control	Cardinality
*Constraint prompts*	*Reasons, goals, aims, objectives*	*Criteria, measures, effectiveness*	*Activities, processes*	*Limitations, capabilities*	*Geographical features*
Trip Purpose (Level 1)	Grocery shopping Church Touring Multiple errands Visiting family and friends Volunteer work Rides for family and friends Bank work				
Travel Conditions (Level 2)		Safety Travel time Less pedestrians Less traffic Direct route Time of day Access to parking			
Driving Challenges (Level 3)			Speeding to keep with flow of the traffic. Crossing a number of wide intersections. Multiple lane changes on 4-lane, high traffic roads. Hard braking for quick turns. Speeding on turns to merge with speeding traffic. Construction zones.		
Physical Properties of the Environment (Level 4)				Onramp Complex navigation Wide intersections High speed limit Crossing 4-lane in traffic Long traffic lights Multiple stop signs Narrow routes Landmarks	
Trip Features (Level 5)					Direction of travel Origin and destination

**Table 7:** Proposed mathematical representation of each of the abstraction hierarchy levels describing route familiarity.

Description of the Abstraction Hierarchy Levels	Driving Context Representation	Mathematical Representation	Similarity Measure
Level 1: Overall purpose, objectives, and *external constraints* on the system due to the environment.	Trip purpose level (e.g., grocery shopping, running errands).	*Similarity* ($S_{F}$) of the trip purpose ($S_{p}$), objectives ($S_{o}$), and external constraints ($S_{e}$) between two or more trips.	$S_{F}=f\left(\begin{array}{c} S_{p},\\ \;S_{o},\;S_{e} \end{array}\right)$
Level 2: Values and priority measures needed to fulfill the functional purpose of the system.	Travel conditions level (e.g., shortest route, fastest route).	*Similarity* ($S_{A}$) in the overlap (O) between the travel conditions criteria (C) between two or more routes.	$S_{A}=f\left(O\left(C\right)\right)$
Level 3: Functions and processes that must be supported to fulfill the system’s functional purpose, independent of the underlying physical objects or object-related processes needed to implement them.	Driving challenges level (e.g., braking, accelerating).	*Similarity* ($S_{G}$) in the overlap of driving challenges criteria (G) between two or more routes.	$S_{G}=f\left(O\left(G\right)\right)$
Level 4: Object-related processes or parts of the system that are used to characterize the functional states.	Physical properties of the environment level (e.g., speed bumps, U-turns).	*Similarity* ($S_{Pfunction}$) in the overlap (M) of sequence of the object-related processes or parts (*N*) between two or more routes.	$S_{Pfunction}=f\left(\begin{array}{c} N,\;\\ M \end{array}\right)$
Level 5: Physical appearance and configuration of the system and its parts.	Trip features level (e.g., home to grocery store).	*Similarity* ($S_{Pform}$) in the overlap (O) of physical location (A), and spatial distribution (Pr) between two or more routes.	$S_{Pform}=f\left(\begin{array}{c} O(A,\;\\ Pr) \end{array}\right)$

## Data Availability

No data are available for sharing.

## References

[1] McGinty L., Smyth B. (2000). Personalised Route Planning: A Case-Based Approach. European Workshop on Advances in Case-Based Reasoning.

[2] Mourant R.R., Rockwell T.H. (1970). Mapping eye-movement patterns to the visual scene in driving: An exploratory study. Hum. Factors.

[3] Allen R.W., Stein A.C., Rosenthal T.J., Ziedman D., Torres J.F., Halati A. A Human Factors Simulation Investigation of Driver Route Diversion and Alternate Route Selection using In-Vehicle Navigation Systems. Proceedings of the Vehicle Navigation and Information Systems Conference.

[4] Kantowitz B.H., Hanowski R.J., Kantowitz S.C. (1997). Driver Acceptance of Unreliable Traffic Information in Familiar and Unfamiliar Settings. Hum. Factors J. Hum. Factors Ergon. Soc..

[5] Lotan T. (1997). Effects of familiarity on route choice behavior in the presence of information. Transp. Res. Part C Emerg. Technol..

[6] Beijer D., Smiley A., Eizenman M. (2004). Observed driver glance behavior at roadside advertising signs. Transp. Res. Rec..

[7] Srinivas S., Hirtle S.C. (2006). Knowledge based Schematization of Route Directions. International Conference on Spatial Cognition.

[8] Martens M.H., Fox M. (2007). Does road familiarity change eye fixations? A comparison between watching a video and real driving. Transp. Res. Part F Traffic Psychol. Behav..

[9] Uc E.Y., Rizzo M., Anderson S.W., Sparks J.D., Rodnitzky R.L., Dawson J.D. (2007). Impaired navigation in drivers with Parkinson’s disease. Brain.

[10] Mader M., Bresges A., Topal R., Busse A., Forsting M., Gizewski E.R. (2009). Simulated car driving in fMRI-Cerebral activation patterns driving an unfamiliar and a familiar route. Neurosci. Lett..

[11] Yanko M.R., Spalek T.M. (2013). Route familiarity breeds inattention: A driving simulator study. Accid. Anal. Prev..

[12] Li D., Miwa T., Morikawa T. (2013). Use of Private Probe Data in Route Choice Analysis to Explore Heterogeneity in Drivers’ Familiarity with Origin-Destination Pairs. Transp. Res. Rec..

[13] Ramachandran D., Karpov I.V., Gupta R., Raux A. Driver Familiarity Modeling for Generating Navigation Directions. Proceedings of the 16th International IEEE Conference on Intelligent Transportation Systems (ITSC 2013).

[14] Marquez D.X., Hunter R.H., Griffith M.H., Bryant L.L., Janicek S.J., Atherly A.J. (2017). Older adult strategies for community wayfinding. J. Appl. Gerontol..

[15] Payyanadan R.P., Lee J.D. (2018). Influence of familiarity on the driving behavior, route risk, and route choice preferences of older drivers. IEEE Trans. Hum.-Mach. Syst..

[16] Intini P. The Impact of Route Familiarity on Drivers’ Speeds, Trajectories and Risk Perception. Proceedings of the 17th International Conference Road Safety On Five Continents (RS5C 2016).

[17] Reimer B., D’Ambrosio L.A., Coughlin J.E., Kafrissen M.E., Biederman J. (2006). Using self-reported data to assess the validity of driving simulation data. Behav. Res. Methods.

[18] Yonelinas A.P. (2002). The nature of recollection and familiarity: A review of 30 years of research. J. Mem. Lang..

[19] Yonelinas A.P. (1994). Receiver-operating characteristics in recognition memory: Evidence for a dual-process model. J. Exp. Psychol. Learn. Mem. Cognit..

[20] Jacoby L.L. (1991). A process dissociation framework: Separating automatic from intentional uses of memory. J. Mem. Lang..

[21] Bonsall P., Firmin P., Anderson M., Palmer I., Balmforth P. (1997). Validating the results of a route choice simulator. Transp. Res. Part C Emerg. Technol..

[22] Hamed M.M., Abdul-Hussain A.A. (2001). Driver’s familiarity with urban route network layout in Amman, Jordan. Cities.

[23] Dia H. (2002). An agent-based approach to modelling driver route choice behaviour under the influence of real-time information. Transp. Res. Part C Emerg. Technol..

[24] Patel K., Chen M.Y., Smith I., Landay J.A. Personalizing Routes. Proceedings of the 19th Annual ACM Symposium on User Interface Software and Technology.

[25] Zhang L., Levinson D. (2008). Determinants of Route Choice and Value of Traveler Information: A Field Experiment. Transp. Res. Rec..

[26] Lin I.C., Chou S.Y. Developing Adaptive Driving Route Guidance Systems based on Fuzzy Neural Network. Proceedings of the Intelligent Environments, 2008 IET 4th International Conference.

[27] Prato C.G., Bekhor S., Pronello C. (2012). Latent variables and route choice behavior. Transportation.

[28] Prato C.G. (2009). Route choice modeling: Past, present and future research directions. J. Choice Model..

[29] Payyanadan R.P., Gibson M., Chiou E., Ghazizadeh M., Lee J.D. (2017). Contextual Design for Driving: Developing a Trip-planning Tool for Older Adults. Transp. Res. Part F Traffic Psychol. Behav..

[30] Papinski D., Scott D.M., Doherty S.T. (2009). Exploring the route choice decision-making process: A comparison of planned and observed routes obtained using person-based GPS. Transp. Res. Part F Traffic Psychol. Behav..

[31] Ittelson W.H. (1976). Environment perception and contemporary perceptual theory. Environmental Psychology: People and Their Physical Settings.

[32] Timpf S. (1999). Abstraction, Levels of Detail, and Hierarchies in Map Series. International Conference on Spatial Information Theory.

[33] Hirtle S.C., Jonides J. (1985). Evidence of hierarchies in cognitive maps. Mem. Cognit..

[34] Smith E.E., Medin D.L. (1989). Categories and Concepts.

[35] Rosch E., Mervis C.B., Gray W.D., Johnson D.M., Boyes-Braem P. (1976). Basic objects in natural categories. Cogn. Psychol..

[36] Tversky B., Hemenway K. (1984). Objects, parts, and categories. J. Exp. Psychol. Gen..

[37] Goldberg L.R. (1986). The validity of rating procedures to index the hierarchical level of categories. J. Mem. Lang..

[38] Vicente K.J., Wang J.H. (1998). An ecological theory of expertise effects in memory recall. Psychol. Rev..

[39] Naikar N. (2013). Work Domain Analysis: Concepts, Guidelines, and Cases.

[40] Hajdukiewicz J.R., Burns C.M., Vicente K.J., Eggleston R.G. Work Domain Analysis for Intentional Systems. Proceedings of the Human Factors and Ergonomics Society 43rd Annual Meeting.

[41] Miller C.A., Vicente K.J. Toward an Integration of Task-and Work Domain Analysis Techniques for Human-Computer Interface Design. Proceedings of the Human Factors and Ergonomics Society Annual Meeting.

[42] Rasmussen J. (1986). Information Processing and Human-Machine Interaction: An Approach to Cognitive Engineering.

[43] Burns C.M., Vicente K.J. (2001). Model-Based Approaches for Analyzing Cognitive Work: A Comparison of Abstraction Hierarchy, Multilevel Flow Modeling, and Decision Ladder Modeling. Int. J. Cogn. Ergon..

[44] Kesting A., Treiber M., Helbing D. (2010). Enhanced intelligent driver model to access the impact of driving strategies on traffic capacity. Philos. Trans. A. Math. Phys. Eng. Sci..

[45] Rasmussen J. (1994). Taxonomy for Work Analysis. Design of Work and Development of Personnel in Advanced Manufacturing.

[46] Donorfio L.K.M., Mohyde M., Coughlin J., D’Ambrosio L. (2008). A Qualitative Exploration of Self-Regulation Behaviors among Older Drivers. J. Aging Soc. Policy.

[47] Myers A.M., Paradis J.A., Blanchard R.A. (2008). Conceptualizing and Measuring Confidence in Older Drivers: Development of the Day and Night Driving Comfort Scales. Arch. Phys. Med. Rehabil..

[48] Ruechel S., Mann W.C. (2005). Self-regulation of driving by older persons. Phys. Occup. Ther. Geriatr..

[49] Helgoe R.S. (1976). Frequency and Recency in Visual Recognition. Psychol. Rec..

[50] Hintzman D.L. (2001). Judgments of frequency and recency: How they relate to reports of subjective awareness. J. Exp. Psychol. Learn. Mem. Cognit..

[51] Zhang J., Ghorbani A.A. Familiarity and trust: Measuring familiarity with a web site. Proceedings of the Second Annual Conference on Privacy, Security and Trust.

[52] Vrotsou K., Forsell C. (2011). A Qualitative Study of Similarity Measures in Event-Based Data. Symposium on Human Interface.

[53] Wixted J.T. (2007). Dual-process theory and signal-detection theory of recognition memory. Psychol. Rev..

[54] Bryden K.J., Charlton J.L., Oxley J.A., Lowndes G.J. (2013). Self-reported wayfinding ability of older drivers. Accid. Anal. Prev..

[55] Lind M. Making Sense of the Abstraction Hierarchy. Proceedings of the Seventh European Conference on Cognitive Science Approaches to Process Control.

[56] Jacko J.A. (2012). Human Computer Interaction Handbook: Fundamentals, Evolving Technologies, and Emerging Applications.

[57] van Otterlo M. (2009). The Logic of Adaptive Behavior.

[58] Vicente K.J., Rasmussen J. (1992). Ecological Interface Design: Theoretical Foundations. IEEE Trans. Syst. Man Cybern..

[59] Laran J., Wilcox K. (2011). Choice, Rejection, and Elaboration on Preference-Inconsistent Alternatives. J. Consum. Res..

[60] Trick L. (2004). Driving and Selective Attention: A Conceptual Framework for Understanding the Role of Selective Attention in Driving.

[61] Johnson E.J., Hardie B.G.S., Meyer R.J., Walsh J. (2006). Observing Unobserved Heterogeneity: Using Process Data to Enhance Choice Models.

[62] Zheng Y., Xie X. (2011). Learning travel recommendations from user-generated GPS traces. ACM Trans. Intell. Syst. Technol..

